# Integrating group antenatal care into routine services: a registry-based cohort study in Geita, Tanzania

**DOI:** 10.1186/s44263-026-00243-4

**Published:** 2026-02-02

**Authors:** Augustino Hellar, Raymond Bandio, Edwin Ernest, Ahmad Makuwani, Alen Kinyina, Phineas Sospeter, Hamid Mandali, Yusuph Kulindwa, Isaac Lyatuu, Wilfred Kafuku, Frank Phiri, Cyprian Mtani, James Tumaini Kengia, Omari Sukari, Husna Athumani, James Hellar, Ntuli Kapologwe

**Affiliations:** 1 Prime Health Initiative Tanzania (PHIT), Mbezi Beach, P.O Box 60011, Kinondoni, Dar-Es-Salaam, Tanzania; 2 Jhpiego Tanzania, An affiliate of the Johns Hopkins University, Dar-Es-Salaam, Tanzania; 3 Ministry of Health Tanzania, Dodoma, Tanzania; 4 Prime Minister’s Office - Regional Authority and Local Government (PMO-RALG), Dodoma, Tanzania; 5 University of Dodoma, Dodoma, Tanzania; 6 Geita Regional Health Secretariat, Prime Minister’s Office - Regional Administration and Local Government (PMO-RALG), Government of the United Republic of Tanzania, Geita, Tanzania; 7 East, Central and Southern Africa Health Community (ECSA-HC) Secretariat, Arusha, Tanzania

**Keywords:** Group antenatal care, ANC4 +, IPTp3 +, Maternal health services, Routine public facilities

## Abstract

**Background:**

Group antenatal care (G-ANC) is an emerging service delivery model that integrates clinical assessments, health education, and peer support within group sessions. While evidence supports its effectiveness in pilot settings, less is known about its feasibility and impact when integrated into routine public health systems in low-resource settings.

**Methods:**

We conducted a registry-based observational cohort study (January 2023–November 2024) across six government facilities in Geita Region, Tanzania (two hospitals, two health centers, and two dispensaries). Pregnant women with gestational age ≥ 20 weeks were enrolled into G-ANC cohorts. Data was abstracted from routine antenatal care (ANC), labor and delivery, and cohort records. Descriptive statistics summarized ANC attendance and service uptake; associations with adverse birth outcomes were examined using a binomial-logit generalized linear mixed model (GLMM).

**Results:**

A total of 5936 women in 149 cohorts were enrolled. Overall, 93.9% completed ≥ 4 ANC visits (ANC4 +); 76.1% received ≥ 3 doses of malaria intermittent preventive treatment in pregnancy with sulfadoxine–pyrimethamine (IPTp3 +); 92.6% received iron–folate supplementation; and 96.2% delivered in health facilities. Only 8.5% initiated ANC in the first trimester, consistent with the ≥ 20-week threshold for G-ANC entry. In multivariable GLMM, completing ≥ 4 ANC visits was associated with lower odds of adverse birth outcomes (adjusted odds ratio [aOR] = 0.122, 95% CI 0.06–0.24; *p* < 0.001). Attendance at hospital-level facilities was associated with higher odds (aOR = 2.91, 95% CI 1.37–6.18; *p* = 0.005), likely reflecting referral of high-risk pregnancies. First-trimester initiation showed no significant association (aOR = 1.04, 95% CI 0.27–3.93).

**Conclusions:**

In routine settings, G-ANC was associated with high ANC attendance, strong uptake of essential interventions, and positive birth outcomes. Integration appears feasible within public systems, though adaptations are needed to promote earlier initiation and alignment with the WHO eight-contact model. Further research should examine costs, feasibility, scalability, and long-term impact.

**Supplementary Information:**

The online version contains supplementary material available at 10.1186/s44263-026-00243-4.

## Background

Antenatal care (ANC) is a critical component of maternal healthcare that ensures the well-being of both the mother and the unborn child. Traditional ANC models typically involve one-to-one consultations between pregnant women and healthcare providers. Group antenatal care (G-ANC), which brings together women of similar gestational age for shared visits, integrates clinical assessments, health education, and peer support in a group setting, fostering increased engagement and improving the overall pregnancy experience [1].

Tanzania has adopted the World Health Organization (WHO) guidelines recommending eight ANC contacts to ensure comprehensive care throughout pregnancy [2]. However, recent data show that only 65% of pregnant women attend at least four ANC visits (ANC4 +) [3]. Tanzania Ministry of Health (MOH) facility-level tools still track ANC4 +, and data on frequency of 8 ANC visits is generally scarce. Recognizing the potential of G-ANC in enhancing ANC services, including the number of ANC contacts, the WHO has recommends it as an alternative to individual ANC in settings where women prefer it and adequate infrastructure exists [4].

Studies across diverse settings report advantages of G-ANC, including improved maternal knowledge, stronger peer support, enhanced client-provider relationships, and higher attendance rates [5–8]. In Kenya, Nigeria, Tanzania, and Malawi, G-ANC has also been linked to increased adherence to preventive interventions such as intermittent preventive treatment for malaria with 3 doses of sulfadoxine-pyrimethamine (SP) (IPTp3 +), improved awareness of danger signs, and higher satisfaction with quality of care [9–14]. Healthcare providers facilitating G-ANC often report enhanced professional satisfaction, highlighting more meaningful interactions with pregnant women and a more holistic approach to maternal services [15].

Yet, most evidence to date comes from pilot projects or research-intensive environments. There is limited data on the effectiveness and integration of G-ANC into routine public sector systems, especially in low-resource settings. Implementation challenges including staffing shortages and infrastructure constraints have been documented in countries like Nepal [16], and slow adoption across LMICs has been attributed to logistical and policy barriers [17]. Despite these challenges, systematic reviews suggest that G-ANC can be effectively adapted for resource-limited environments when contextualized [18, 19]. In Tanzania, a prior assessment in Geita showed that only 34% of pregnant women completed ANC4 + visits, and 19% initiated care in the first trimester [20]. This study presents an endline evaluation of G-ANC implementation outside of pilot or research settings.

Grounded in the WHO 2016 ANC framework [4] and implementation science principles, this study examines the integration of G-ANC within routine public health systems, drawing on data from nearly 6000 women who received ANC in participating facilities over a 20-month period, thus providing insights into integration, uptake, and service utilization of the G-ANC model in routine care. We mainly sought to (i) describe the implementation process of G-ANC as guided by the WHO ANC framework and adopted following the Jhpiego G-ANC approach [1]; (ii) examine service utilization patterns, particularly ANC attendance and uptake of preventive interventions, among women enrolled in G-ANC; and, finally, (iii) assess birth outcomes, across six public health facilities in Geita region, Tanzania.

## Methods

### Study design and setting

We conducted a quantitative registry-based observational cohort study, nested within a government-endorsed G-ANC implementation initiative (Mlinde Mama Project), conducted across six public health facilities in Geita Region, Tanzania, from January 2023 to November 2024. The Mlinde Mama Project was a 2-year initiative to develop and pilot an artificial intelligence-enabled digital platform to support ANC by predicting complications, guiding clinical management, and improving outcomes, using G-ANC as the primary entry point (https://phit.or.tz/mama.php).

The six facilities included two hospitals, two health centers, and two dispensaries distributed across Geita Region, in the Northwestern part of Tanzania. Together, these facilities serve both peri-urban and rural populations, with immediate catchment areas ranging from 25,000 to 100,000 residents each. Monthly ANC attendance varied across facilities, ranged between 370 and 500 at the dispensaries, 400 and 600 at the district hospitals, and 1400 and 1600 at the health centers (health centers had the highest ANC volume). During the 20-month implementation period, a total of 5936 pregnant women were enrolled into 149 G-ANC cohorts. Staff–client ratios also differed by facility type: hospitals generally staffed 4–6 skilled providers per ANC clinic day, health centers 2–4, and dispensaries 1–2. To support implementation, thirty health workers (nurses, midwives, clinical officers) and six regional Trainers of Trainers (ToT) were trained on the G-ANC model. Our reporting adheres to the Strengthening the Reporting of Observational Studies in Epidemiology (STROBE) [21] and Standards for Reporting Implementation Studies (StaRI) guidelines [22]. Completed StaRI and STROBE checklists are included as Supplementary Material 1 and 2*.*

Facilities were selected using a structured readiness assessment (Supplementary Material 3) developed by the Mlinde Mama team. The assessment combined direct observation, record review, and interviews with facility in-charges and considered the following key aspects:

- Staff availability: number and cadre of skilled ANC providers present on clinic days and availability of trainers or mentors who are knowledgeable on ANC or facility access to district-level trainers/mentors.
- Supportive infrastructure: availability of adequate space to host group sessions of 10–15 women, adjacent private rooms for individual assessments, essential equipment for ANC provision (BP machines, scales, fetoscopes, Hb testing device, urinalysis, HIV/syphilis rapid test kits, data storage capacity, infection prevention supplies, and WASH (water, sanitation, and hygiene).
- Facility-level commitment: engagement of facility leadership and district reproductive health coordinators, willingness to allocate staff time and space for G-ANC sessions, and prior experience with quality improvement initiatives.

Facilities meeting minimum thresholds (routine ANC services offered, available space for group sessions, availability of basic functional ANC equipment, and leadership endorsement) were selected. These were hospitals: Chato and Nzera; health centers: Bwanga and Katoro; and dispensaries: Nkome and Butengorumasa.

### Implementation strategy and framework

**Fig. 1:**
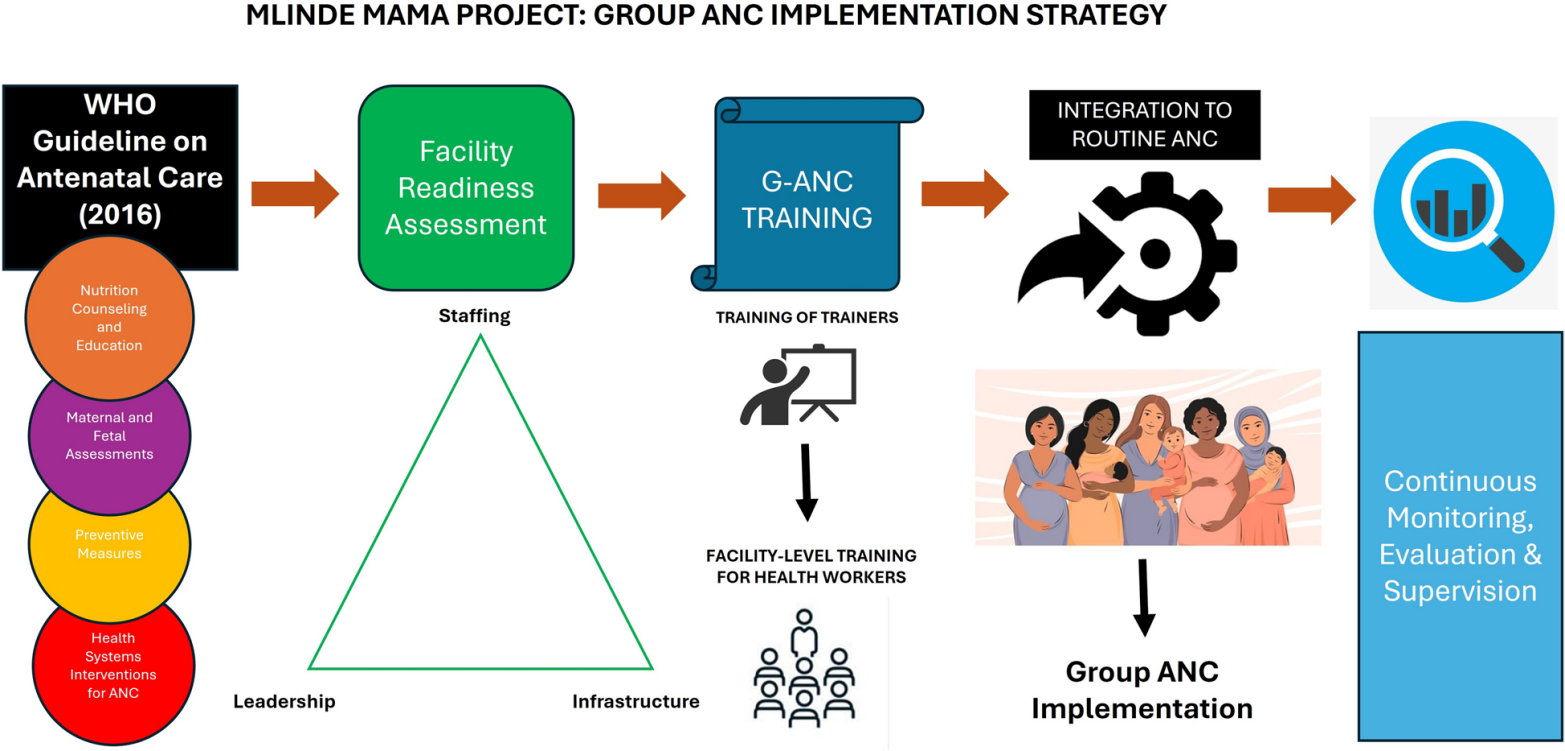
Implementation framework for G-ANC. Legend: Conceptual sequence for integrating G-ANC into routine services in Geita. Steps: (1) alignment with the WHO 2016 ANC guideline; (2) facility readiness assessment across staffing, infrastructure, and leadership; (3) ToT training followed by facility-level training; (4) integration of G-ANC sessions into routine ANC; and (5) continuous monitoring, evaluation, and supervision to support fidelity and adaptation (framework developed for the Mlinde Mama Project)

Implementation followed a structured, government-led strategy to maximize ownership and sustainability, and based on the 2016 WHO ANC recommendations (Fig. 1). Our implementation strategy emphasized integration into existing ANC services rather than creating parallel systems. We rolled out training to front-line facility-level health providers using the six trained ToTs.

Facility leadership was engaged from the outset, and district reproductive health coordinators provided oversight. Supportive supervision, periodic mentoring, and joint data reviews ensured fidelity and adaptation during rollout. The combination of readiness assessment, government ownership, capacity-building, and supervision constituted the core implementation strategy.

The implementation framework was developed by the study team to guide the introduction of G-ANC in Geita Region. The framework illustrates the sequential steps: aligning with WHO ANC guidelines, assessing facility readiness (staffing, infrastructure, leadership), cascading training, integrating G-ANC into routine ANC workflows, and continuous monitoring, evaluation, and supervision.

### Intervention description

G-ANC followed a standardized, WHO aligned model. Pregnant women with a similar gestational age (≥ 20 weeks) were assigned into fixed G-ANC cohorts of 8–12 women. Cohort membership was closed: once formed, women remained in the same group throughout the remainder of their ANC period. Each cohort participated in structured sessions integrating clinical assessments, health education, and peer support. The education curriculum covered topics such as maternal nutrition, malaria prevention, birth preparedness, danger signs, and postpartum care. Recruitment into G-ANC cohorts concluded in mid-June 2024 to ensure that all enrolled participants could be followed through to delivery by the study’s endline in November 2024.

Individual ANC continued in parallel with G-ANC throughout the study period. Women declining group sessions were managed through routine one-to-one visits, ensuring equity and continuity of care.

The project team, in collaboration with subject matter experts from MOH, the Prime Ministers’ Office (PMO-RALG) and Geita regional authorities, adapted the G-ANC materials from Jhpiego’s G-ANC pilot model. Jhpiego is an international non-profit affiliate of Johns Hopkins University (headquartered in Baltimore, MD, USA, with a local country office in Dar es Salaam, Tanzania) that pioneered early pilot implementation of G-ANC across the globe. The adapted G-ANC package comprised five monthly, 2-h meetings facilitated by clinical officers, nurses, and midwives, with CHW support (Table 1). For session three, male partners were invited. However, cohorts were not split by partner attendance and partner attendance was not systematically recorded.

**Table 1:** G-ANC meeting schedule and assessments

Meetings/visits	Timing (GA in weeks)	Theme/topic	Key investigations
Entry point (1st ANC visit)	All women presenting for ANC for the first time (regardless of their GA)	• General ANC counselling • Information on availability of G-ANC services • Opportunity to participate in the G-ANC intervention if willing • If consenting (verbal), allocation to appropriate G-ANC cohort: o < 20 weeks Give appointment for next visit o ≥ 20 weeks schedule for Meeting 1	• Pregnancy test for confirmation (if needed) • USS (if indicated) • Full blood count/hemoglobin, • Urinalysis • Tobacco/substance abuse, GBV screening
Meeting 1 (2nd ANC visit)	20–23 weeks	• Registration • Introduction to group care • Preventing problems in pregnancy • Pairing for peer-to-peer assessments	• Full blood count/hemoglobin, • Urinalysis • HIV test • TB screening • Proteinuria • Syphilis testing • Tobacco/substance use, GBV screening
Meeting 2 (3rd ANC visit)	24–27 weeks	• Recognizing problems in pregnancy • Labor signs: what to expect in labor and birth • Joint planning on topics for male engagement in meeting 3	• Full blood count/hemoglobin • Urinalysis sugar • Tobacco/substance use, GBV screening
Meeting 3 (4th ANC visit) male partners invited to participate)	28–31 weeks	• Partner’s role in a positive pregnancy experience • Birth preparedness and complications readiness • Healthy timing and spacing of pregnancies • Gender-based violence • Visit to the labor ward	• Full blood count/hemoglobin, • Urinalysis • HIV re-test • TB screening • Tobacco/substance use, GBV screening
Meeting 4 (5th ANC visit)	32–35 weeks	• Preventing problems after birth • Contraceptive methods include LAM	• Full blood count/hemoglobin • Proteinuria • Tobacco/substance use, GBV screening
Meeting 5 (6th ANC visit)	36–40 weeks	• Support during labor • Recognizing problems in the newborn • Recognizing maternal problems after birth • Birth planning	• Full blood count/hemoglobin • Blood Group + Rh Factor • Tobacco/substance use, GBV screening

Definitions: *GBV* gender-based violence, *LAM* lactational amenorrhea, *Rh Factor* rhesus factor, *TB* tuberculosis, *USS* ultrasound scan

### Study population and data sources

The study population included all pregnant women who enrolled in G-ANC cohorts at the six study facilities during the implementation period and had complete ANC and delivery records. A total of 5936 women in 149 unique cohorts met these criteria and were included in the analysis. Women who did not opt into G-ANC were offered routine individual ANC without prejudice or compromise in the standard of care. Outcome data were extracted from ANC registers, cohort tracking tools, and labor/delivery records using a standardized abstraction form by trained data clerks. All pregnant women who were eligible (gestational age ≥ 20 weeks) and opted to enroll in one of the 149 G-ANC cohorts during the 20-month implementation period were included in the study. This study presents the full census of all 5936 women, not a sampled subset.

By design, formal entry into G-ANC required gestational age ≥ 20 weeks. Women presenting before 20 weeks received routine individual ANC in line with national guidelines and were subsequently transitioned into their G-ANC cohort once they reached 20 weeks. Therefore, while ANC register data captured some women presenting in the first trimester, their exposure to the G-ANC intervention began only at ≥ 20 weeks. This distinction is important when interpreting gestational age at first contact versus timing of G-ANC entry. The study dataset has been deposited in the Harvard data repository [23].

### Projected implementation target

No formal sample-size calculation was performed. Instead, we projected an implementation target of 6000 women based on client flow and facility capacity across the six participating facilities. This projection considered expected ANC volumes, the availability of trained health providers, and the physical space required to conduct group sessions. Initial projections suggested that up to 8800 women could be eligible based on national and regional estimates of ANC initiation over the 20-month implementation period. This figure represented approximately 47% of the total expected ANC client workload, implying an estimated total of 18,700 clients across the participating facilities during the study period. However, practical constraints such as staffing and session capacity necessitated revising this figure to a feasible target of 6000 G-ANC participants. All eligible women attending ANC at the study facilities during the implementation period were invited to participate in G-ANC.

### Variables and outcomes

#### Primary and secondary endpoints

The primary endpoint for our study was completion of ≥ 4 ANC visits, defined as the proportion of women recorded with at least four contacts across G-ANC sessions and individual ANC visits during pregnancy. Secondary endpoints included (a) completion of IPTp3 +, defined as receiving ≥ 3 doses of intermittent preventive treatment for malaria with SP: receipt of at least three documented doses of SP in the ANC register; (b) facility delivery: birth documented in the facility register as having occurred in a health facility; and (c) adverse birth outcomes: stillbirth, defined as birth with no signs of life at ≥ 28 + 0 weeks’ gestation (or, if GA was missing, birthweight ≥ 1000 g or length ≥ 35 cm). Fresh stillbirth (FSB) is intrapartum death at ≥ 28 + 0 weeks’ gestation with no maceration; macerated stillbirth (MSB) indicates antepartum death (maceration present). Intra-uterine fetal death (IUFD) refers to any fetal death in utero prior to delivery. Outcomes were abstracted as recorded by midwives in routine labor/delivery registers. Independent variables included maternal age and gestational age at enrollment.

### Data collection procedures

After 20 months of study implementation, data were abstracted retrospectively, by trained data clerks, from routine facility records, including MOH Hospital Information Management System (HMIS) registers (ANC and labor & delivery registers), and a study cohort tracker. We used electronic data abstraction forms developed through an open-source online software, KoboToolBox [24]. The forms were pretested and standardized across all sites. Facility staff and data clerks were trained in proper documentation and abstraction protocols.

### Data quality assurance

To ensure data accuracy, quality control checks were conducted through periodic supervisory visits during the study implementation period. During data collection, supervisors validated data entries against source documents and resolved inconsistencies through discussion with facility staff. To further enhance reliability, all facility staff and data personnel received standardized training on data abstraction procedures, and inter-rater reliability checks were conducted at selected intervals. These checks involved comparing data independently abstracted from the same records by different data clerks to assess consistency. Discrepancies were discussed and resolved collaboratively.

### Data analysis

Descriptive statistics were used to summarize participant characteristics and service utilization. Data was analyzed using a multivariable generalized linear mixed model (GLMM) to account for the clustered structure of the data, where individual observations were nested within 149 cohorts and 6 facilities. The GLMM was specified using a binomial distribution with a logit link function, with facility ID as a random intercept. The model examined associations between selected ANC predictors and adverse birth outcomes. The dependent variable was birth outcome, categorized as “adverse” (FSB or IUFD) versus “alive without complication.” The independent variables included the following: (i) attending four or more ANC visits, (ii) first-trimester ANC initiation, and (iii) facility level of ANC attendance (hospital or health center, with dispensary as the reference group). Random intercepts were specified for both cohort and facility to account for intra-cluster correlation. Fixed effects for the predictor variables were estimated using maximum likelihood estimation. Results are presented as adjusted odds ratios (aOR) with corresponding 95% confidence intervals (CIs). Statistical significance was assessed at a two-tailed alpha level of 0.05. All analyses were conducted in Python using the binomial Bayesian mixed GLMM framework. We fit the binomial-logit GLMM with random intercepts for cohort and facility to account for clustering. Fixed effects included the following: (i) ANC4 + (yes/no), (ii) first-trimester ANC initiation (yes/no), and (iii) facility level (hospital, health center; reference = dispensary).

Regression analyses were undertaken to explore associations between maternal characteristics and outcomes. These analyses should be interpreted as exploratory, as the study was not powered for hypothesis testing or for detection of specific minimum effect sizes.

For each outcome, the denominator reflects the number of women with available data for that variable. No imputation was applied to missing values.

## Results

A total of 5936 pregnant women were enrolled into G-ANC cohorts across the six public health facilities in Geita Region between January 2023 and August 2024. Pre-specified outcomes include completion of ≥ 4 ANC visits, completion of ≥ 3 doses of IPTp3 + with SP, level of the facility where delivery took place, and adverse birth outcomes (i.e., FSB or IUFD).

The average gestational age at enrollment was 19.9 weeks (SD 3.9). Most participants were aged 20–34 years (Table 2). A total of 149 G-ANC cohorts were established across the six facilities, with cohort sizes varying by site.

**Table 2:** Number of G-ANC clients by facility

Health facility	G-ANC clients
Butengorumasa Dispensary	459
Bwanga Health Center	1265
Chato District Hospital	846
Katoro Health Center	1593
Nkome Dispensary	1082
Nzera District Hospital	691
**Total**	**5936**
**Gestational age, mean (SD)**	**19.9 (3.9)**

The largest number of women was enrolled at Katoro Health Center (*N* = 1593), and the smallest group at Butengorumasa Dispensary (*N* = 459)

The largest number of women were enrolled at Katoro Health Center (*N* = 1593), and the smallest group at Butengorumasa Dispensary (*N* = 459).

### ANC attendance and service utilization

**Fig. 2:**
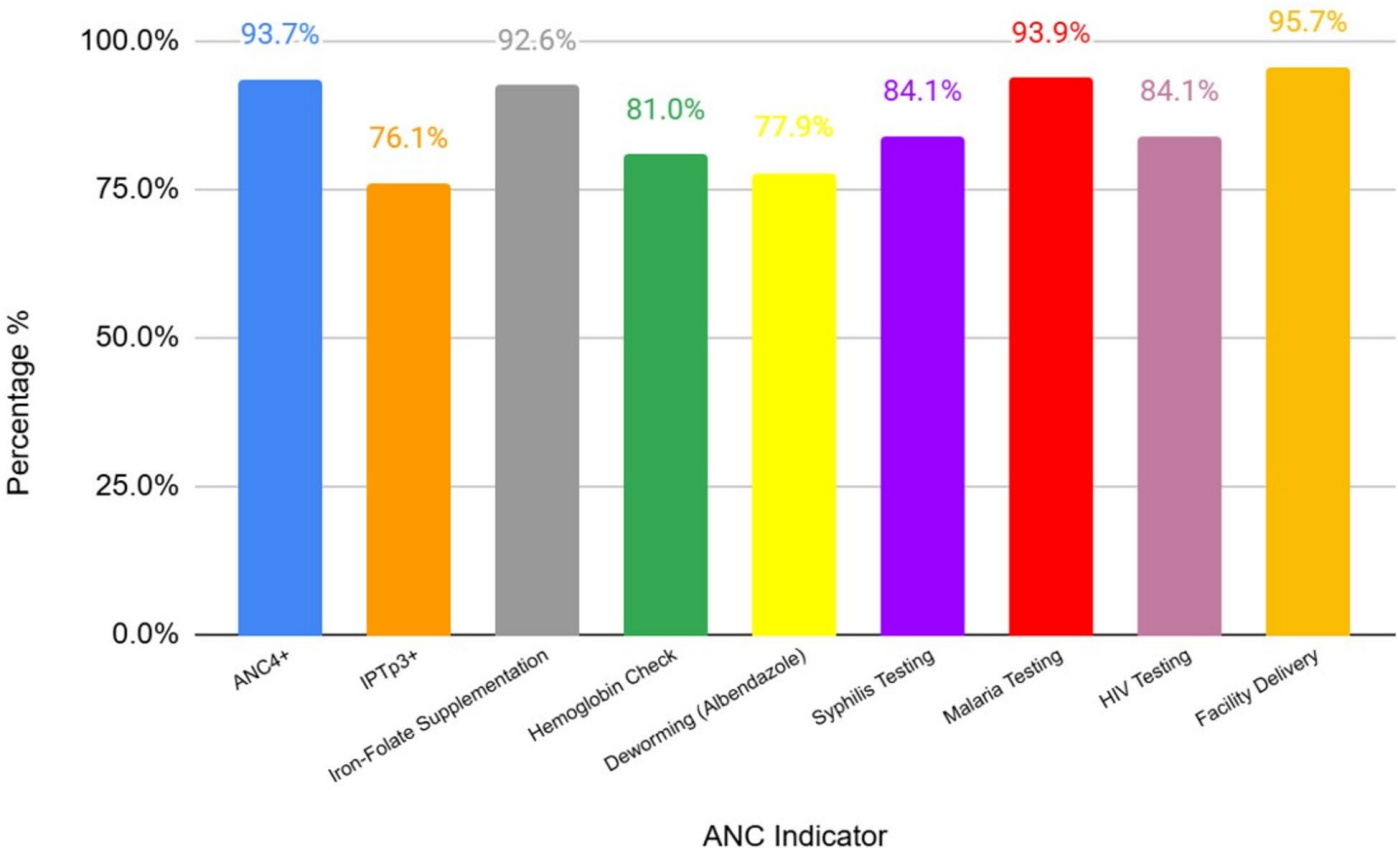
Coverage of key ANC interventions (overall). Legend: Overall coverage of selected ANC indicators among women enrolled in G-ANC across six public facilities in Geita, Tanzania. Bars show the proportion of women meeting each indicator using the total number with available data for that indicator as the denominator. Indicators: completion ANC4 +; receipt of IPTp3 +; iron–folate supplementation; Hb check; deworming with albendazole; syphilis testing; malaria testing; HIV testing; and facility delivery. Data were abstracted from routine registers; no imputation was performed

**Fig. 3:**
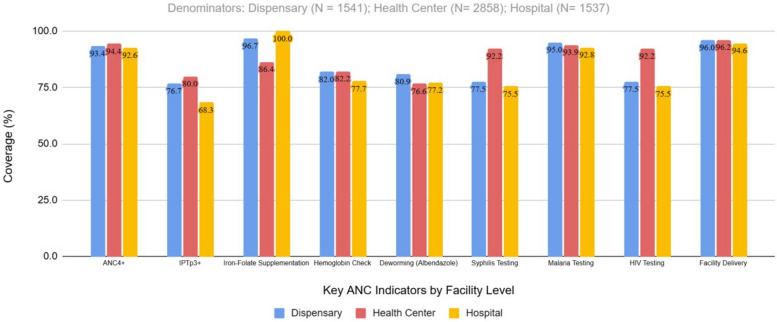
Coverage of key ANC services by facility level. Legend: Facility-level coverage of the same ANC indicators shown in Fig. 2, stratified by level of care. Facility-specific denominators were used for each indicator: dispensary (*N* = 1541); health center (*N* = 2858); hospital (*N* = 1537). Each bar represents the proportion of women at that facility level who met the indicator using routine data available for that indicator. Indicators include ANC4 +, IPTp3 +, iron-folate supplementation, hemoglobin testing, deworming (albendazole), syphilis testing, malaria testing, HIV testing, and facility delivery. The percentage of women within each facility level who received the indicated ANC service (dispensary, health center, hospital) is calculated as coverage = achieved ÷ denominator × 100 for each facility level

Among all G-ANC participants, 93.9% (5573/5936) attended four or more ANC visits (including both group and non-group contacts). IPTp3 + coverage was 76.1% (4517/5936). 92.6% (5497/5936) of women received iron–folate supplementation and 77.9% (4623/5936) received deworming treatment. Majority (96.2% 5682/5936) of the women participating in the G-ANC model delivered at a health facility (Fig. 2). Coverage of ANC utilization by facility level is presented in Fig. 3.

### GANC enrollment by gestation age

**Fig. 4:**
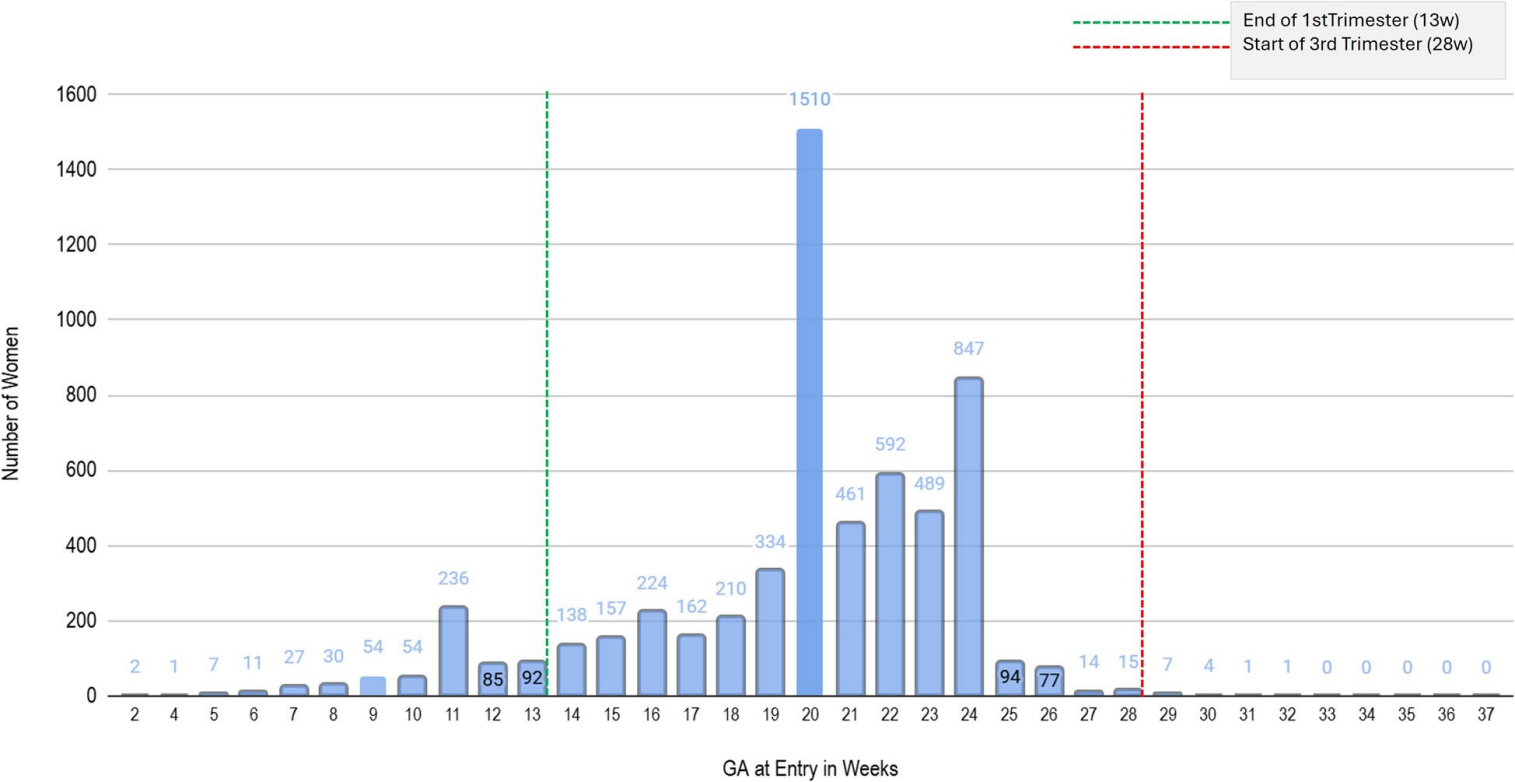
Gestational age at entry into G-ANC, by trimester. Legend: Distribution of GA in weeks at first recorded G-ANC session among enrolled women. Bars display the proportion of all enrolled women entering at each week of gestation. Vertical annotations mark the end of the first trimester (13 weeks) and the start of the third trimester (28 weeks). In this program, women initiating ANC before 20 weeks received routine individual care and were invited to formally join G-ANC at ≥ 20 weeks, which contributes to the concentration of entries in the second trimester. A total of 507/5936 women (8.5%) initiated ANC during the first trimester, 5309/5936 women (89.4%) during the second, and 28/5936 women (0.47%) came for the first ANC visit during the third trimester

A total of 507/5936 women (8.5%) initiated ANC during the first trimester, 5309/5936 women (89.4%) during the second, and 28/5936 women (0.47%) came for the first ANC visit during the third trimester (see Fig. 4).

### ANC visits by facility level of care

Among the 5936 pregnant women enrolled in the study, nearly half (48.1%) received ANC at health centers, while 26.0% attended dispensaries and 25.9% attended hospitals. Overall, 93.9% of participants completed ANC4 + visits, with minor variation by facility type. None of the women in this cohort achieved the WHO-recommended eight ANC contacts; the maximum observed was six visits. This reflects the design of the five-session adapted G-ANC model as summarized in Table 1.

### Multivariate analysis of birth outcomes

Multivariate logistic regression was conducted to explore associations between selected predictors and adverse birth outcomes (FSB or IUFD).

Using GLMM, which accounted for clustering by cohort and facility, ANC4 + was strongly associated with a reduction in adverse birth outcomes (FSB and IUFD). Women who attended four or more ANC visits had significantly lower odds of adverse birth outcomes (aOR = 0.122, 95% CI 0.06–0.24; *p* < 0.001), indicating a strong protective effect. Attendance at hospital-level ANC was associated with higher odds of adverse birth outcomes compared with lower-level facilities (aOR = 2.91, 95% CI 1.37–6.18). In contrast, attendance at health centers showed a trend toward reduced odds (aOR = 0.26, 95% CI 0.05–1.23), although this did not reach statistical significance (*p* = 0.09). Early initiation of ANC during the first trimester was not significantly associated with adverse birth outcomes (aOR = 1.04, 95% CI 0.27–3.93) (Table 3).

**Table 3:** Distribution of ANC visits by facility level (*N* = 5936)

ANC visits	Dispensary (*N* = 1541)	Health center (*N* = 2858)	Hospital (*N* = 1537)	Total (*N* = 5936)
1–3 visits	91 (5.9%)	161 (5.6%)	111 (7.2%)	363 (6.1%)
4 + visits	1450 (94.1%)	2697 (94.3%)	1426 (92.8%)	5573 (93.9%)
**Total**	**1541 (26%)**	**2858 (48.1%)**	**1537 (25.9%)**	**5936**

The observed differences in the proportion of women attending four or more ANC visits across facility levels (dispensary, health center, hospital) are not statistically significant (chi-square = 4.55, df = 2, *p* = 0.103). This suggests that while there are small variations in ANC4 + attendance between facility types, these differences could be due to chance (note “df” = degrees of freedom)

## Discussion

This implementation study of G-ANC in routine public health facilities in Geita Region, Tanzania, found a high completion rate of ANC4 + and strong uptake of key interventions among nearly 6000 enrolled women. To our knowledge, this is the first large G-ANC implementation study outside of trial settings in Tanzania. Among nearly 6000 women enrolled in G-ANC, 94% completed ANC4 + visits. These findings suggest that G-ANC, when embedded into routine health systems and supported by trained providers, can enhance ANC services uptake even in resource-limited settings. However, it is important to note that we did not systematically document other concurrent facility-level initiatives (e.g., staff training, respectful maternity care programs, infrastructure upgrades) that could have been simultaneously implemented during the study period. Therefore, comparisons with historical baseline data should be interpreted as contextual rather than causal, acknowledging that multiple factors beyond G-ANC may have contributed to observed improvements. The observed ANC4 + completion rate of 94% contrasts favorably with previous national data from Tanzania [3] and earlier data from Geita region [20, 25]. As stated above, these comparisons may be contextual rather than statistical; however, we believe that the findings support prior evidence that the G-ANC model promotes consistent care-seeking through peer encouragement, scheduled visits, and stronger client–provider engagement. Similar studies have reported that the interactive and supportive nature of group sessions enhances women’s motivation to adhere to recommended ANC schedules, thus enhancing overall maternal satisfaction with services [18, 26] and, ultimately, improving maternal and newborn outcomes [10, 17, 27, 28].

Despite these improvements, only 8.5% of G-ANC participants initiated ANC in the first trimester, lower than the 19% observed in the same facilities at baseline [20]. This reduction likely reflects the design of the G-ANC model as explained in the results. Future adaptations should explore mechanisms for integrating women into G-ANC earlier to align with national and international guidelines promoting first-trimester entry.

The IPTp3 + coverage (76.1%) approaches the national target of 80% and represents a notable improvement from the 43% baseline level [20]. This may reflect the structured group education format, where facilitators can emphasize the importance of malaria prevention in pregnancy and ensure continuity across visits. Other similar studies with approaches that promote women’s empowerment and community involvement have reported similar findings [28–32]. However, persistent shortfalls may be linked to known barriers such as intermittent stockouts of SP, provider hesitancy to administer SP at late gestational ages, and client-level misconceptions, factors also reported in other sub-Saharan African contexts [33, 34].

Additionally, there was adequate coverage of key ANC interventions such as iron-folate supplementation, albendazole administration, and screening for HIV, syphilis, malaria, and hemoglobin levels. These integrated services, delivered consistently in G-ANC sessions, likely benefited from the structured nature of group visits and the consistent presence of trained providers. Evidence from previous studies has similarly shown that G-ANC improves both the comprehensiveness and quality of care by facilitating better organization, increased patient-provider interaction, and greater adherence to ANC protocols [9, 14, 28, 35].

Iron-folate supplementation coverage was at 96%. This high rate could reflect the impact of regular health education and peer motivation during G-ANC on the uptake of preventive interventions. Evidence shows that addressing anemia in pregnancy is critical to improving birth outcomes, and G-ANC offers a platform to reinforce messages around adherence and timely supplementation [36, 37].

The proportion of facility-based deliveries in this study was 96%, only a percentage higher than the 95% reported at baseline [20]. This finding is likely due to the already high baseline performance, limiting the measurable scope for further improvement. Nonetheless, maintaining such high rates is essential for reducing maternal and neonatal mortality and requires continued community mobilization, quality service delivery, and monitoring [38].

Despite high overall attendance for ANC across facility levels, our analysis revealed no statistically significant difference in the proportion of women achieving ANC4 + when comparing dispensaries, health centers, and hospitals. This finding suggests that all facility levels, regardless of size or scope, were similarly effective in supporting women to meet recommended ANC attendance thresholds. Although the differences were small, health centers had the highest proportion of ANC4 + attendance, closely followed by dispensaries and then by hospitals, indicating that the G-ANC model may have promoted consistent care-seeking behavior irrespective of facility type. These findings are particularly relevant in low-resource settings where health system capacity varies widely by facility level. The comparable performance of dispensaries and health centers with hospitals in achieving high ANC4 + coverage underscores the potential of decentralized care models like G-ANC to improve maternal health outcomes at scale. The lack of significant disparity reinforces the importance of strengthening ANC services across all facility tiers to ensure equitable and timely access to care. The fact that hospitals compared slightly less favorably in ANC4 + attendance compared to health centers and dispensaries may reflect their referral role, where women present late in pregnancy with complications, limiting opportunities for multiple ANC visits. This similar context has been another study in Tanzania that showed variation on maternal service utilization by facility level [39]. We therefore note that, despite improvements in utilization, our findings highlight some disparities in G-ANC performance across facility levels.

The regression analysis revealed that women attending ANC4 + visits had a substantially reduced the likelihood of experiencing adverse outcomes (FSB or IUFD). This finding aligns with WHO recommendations emphasizing the importance of multiple ANC contacts for timely detection and management of pregnancy-related risks. Conversely, women receiving ANC primarily at hospitals exhibited higher odds of adverse birth outcomes. We hypothesize that both the lower ANC4 + attendance at the hospital level noted above and the higher odds of adverse outcomes at hospitals may reflect a higher workload and referral of more complex cases at this level. Hospitals often manage higher-risk pregnancies referred from lower-level facilities. Therefore, this finding does not necessarily imply that hospital care increases risk but rather that these women represent a higher-risk subgroup. Therefore, our findings call for context-sensitive implementation strategies when introducing new approaches like G-ANC [40, 41].

While G-ANC improved overall attendance, the delayed entry protocol related to the design of the G-ANC model likely limited early initiation. Revisiting this design aspect could enhance the model’s full potential.

This study provides important implementation evidence on G-ANC in routine public health settings, using data from nearly 6000 women across six government-managed facilities. The large sample size, consistent data abstraction procedures, and integration within existing health systems strengthen the relevance and potential generalizability of the findings. Data were drawn from routine service records, reflecting real-world performance under typical health system conditions. However, several limitations must be considered. First, as this was an observational, registry-based cohort study without a comparison group implementation study, hence causal inference is limited. As such, the findings cannot establish whether observed outcomes were a direct result of G-ANC participation. Rather, they describe associations and patterns within the G-ANC population. It is plausible that women who enrolled in G-ANC differed systematically from those who did not, for example, in socioeconomic status, health-seeking behaviors, or baseline health status, and these differences may partly explain the outcomes observed. Therefore, the improvements observed cannot be attributed solely to G-ANC, as concurrent health system changes or external influences may have contributed. Future quasi-experimental design studies where a causal inference is inherent may effectively demonstrate the impact of this model. Second, we did not perform a formal sample size calculation for our study but rather used projected implementation targets. We acknowledge that some wide confidence intervals in Table 4 may reflect limited statistical power and variability in subgroup sizes, rather than absence of an effect. In addition, some outcome estimates, particularly those related to birth complications, may be affected by the referral nature of hospital-level facilities, which typically manage more complex cases. Third, the delayed eligibility for G-ANC (≥ 20 weeks) likely influenced reporting of early ANC initiation, underestimating actual first-trimester contact. Early ANC contacts recorded as individual visits may have contributed to underestimation of first-trimester initiation in our G-ANC dataset. This highlights an important consideration for future adaptations of G-ANC models, where hybrid implementation may influence both measurement and provider workload. Furthermore, first-trimester ANC initiation data were obtained retrospectively from client registers for women enrolled at ≥ 20 weeks. This could introduce potential selection bias due to left truncation. Fourth, feasibility domains such as acceptability and fidelity were not systematically assessed within this analysis. These dimensions would provide a fuller picture of the feasibility of integrating G-ANC into routine public health settings.

**Table 4:** Logistic regression results – predictors of adverse birth outcomes

Predictor	Coef	Standard error	aOR	aOR_95%_CI	*p*-value	Reference
ANC 4 +	− 2.102	0.341	0.122	0.063–0.238	< 0.001	-
ANC first trimester	0.037	0.679	1.037	0.274–3.926	0.957	-
Attend ANC at hospital	1.070	0.383	2.914	1.375–6.177	0.005	Dispensary
Attend ANC at health center	− 1.351	0.796	0.259	0.054–1.233	0.090	Dispensary
Intercept	− 5.325	0.326	0.005	0.003–0.009	< 0.001	-

GLMM with binomial logit link; random intercepts for cohort and facility. Reference category for facility level = dispensary. Variables retained if *p* < 0.10 in univariable analysis or for clinical relevance; adjusted for gestational age at entry (weeks)

*aOR* adjusted odds ratio; *CI* confidence intervals

## Conclusions

The implementation of G-ANC in public health facilities in Geita Region, Tanzania, was associated with improved maternal health service utilization. Among women enrolled in G-ANC, 94% completed ANC4 + contacts, and coverage of IPTp3 +, iron–folate supplementation, and facility-based delivery exceeded national benchmarks. These findings demonstrate that G-ANC can be integrated into routine government ANC services and was linked to improved maternal service utilization in Geita Region.

While IPTp3 + uptake improved compared to previously reported baseline levels, coverage remains slightly below national targets, suggesting the need for strengthened supply chains, provider training, and community education. Facility-level analysis highlighted the importance of mid-level health centers in supporting consistent ANC attendance, while also identifying potential bottlenecks in referral hospitals. These patterns emphasize the need for context-adapted implementation strategies and stronger referral coordination.

This study contributes to the growing evidence base supporting the effectiveness and integration of G-ANC in routine low-resource public health settings. Policymakers and implementers should consider tailoring G-ANC entry criteria to promote earlier initiation and prioritize investments that strengthen lower-level health facilities. Future studies should assess the long-term impact, cost-effectiveness, and sustainability of G-ANC integration within national ANC programs.

## Supplementary Information

Supplementary Material l: StaRI Checklist - Completed Standards for Reporting Implementation Studies checklist with manuscript cross-references.

Supplementary Material 2: STROBE Checklist - Completed STROBE cohort checklist with manuscript cross-references.

Supplementary Material 3: Readiness Assessment Tool - facility readiness assessment instrument to evaluate staffing, infrastructure/equipment, leadership/commitment with scoring guidance.

## Data Availability

The de-identified dataset generated and analyzed during this study, along with the accompanying data dictionary, has been deposited in the Harvard Dataverse repository and is publicly available at: (Hellar et al., 2025; DOI: 10.7910/DVN/RLAJMC) [23]. Data have been fully anonymized in accordance with guidance from MOH, Tanzania, and the National Institute for Medical Research (NIMR). Users are expected to ensure confidentiality and appropriate use of the dataset in line with ethical approvals.

## Abbreviations

ANC: Antenatal care
ANC4 +: At least 4 ANC visits
CHW: Community health worker
DHIS2: District Health Management System (Version 2)
FeFO: Iron (Fe) and folic acid
FSB: Fresh still birth
GA: Gestational age
G-ANC: Group antenatal care
GLMM: Generalized linear mixed model
Hb: Hemoglobin
HMIS: Health Medical Information System
IPTp: Intermittent preventive therapy for malaria with sulfadoxine-pyrimethamine
IPTp3 +: Intermittent preventive therapy for malaria (> 3 doses) with sulfadoxine-pyrimethamine
IUFD: Intrauterine fetal death
LMICs: Low- and middle-income countries
MOH: Ministry of Health
NatREC: National Health Research Ethics Committee
NIMR: National Institute for Medical Research
PMO-RALG: Prime Minister’s Office Regional Authority and Local Government
SP: Sulfadoxine—pyrimethamine
STaRI: Standards for Reporting Implementation Studies
STROBE: Strengthening the Reporting of Observational Studies in Epidemiology
TDHS: Tanzania Demographic Health Survey
TOTs: Trainer of Trainees
WHO: World Health Organization

## References

[1] Suhowatsky S. Group ANC—it’s better together, https://www.jhpiego.org/blog/maternal-and-child-health/group-anc-its-better-together/ (2024, accessed 17 April 2025).

[2] Ministry of Health Tanzania, Community Development, Gender, Elderly and Children. Tanzania antenatal care guidelines, https://platform.who.int/docs/default-source/mca-documents/policy-documents/guideline/tza-mn-21-01-guideline-2018-eng-anc-guidelines.pdf (2018).

[3] Ministry of Health (MoH) [Tanzania Mainland], Ministry of Health (MoH) [Zanzibar], National Bureau of Statistics (NBS), Office of the Chief Government Statistician (OCGS) and ICF. Tanzania demographic and health survey and malaria indicator survey 2022: key indicators report, https://dhsprogram.com/pubs/pdf/FR382/FR382.pdf (2023).

[4] World Health Organization. WHO recommendations on antenatal care for a positive pregnancy experience. Geneva: World Health Organization; 2016, https://www.who.int/publications/i/item/9789241549912 (2016, accessed 14 April 2025). 28079998

[5] Musabyimana A, Lundeen T, Butrick E, et al. Before and after implementation of group antenatal care in Rwanda: a qualitative study of women’s experiences. Reprod Health. 2019;16:90. 31248425 10.1186/s12978-019-0750-5 PMC6595554

[6] Gaur BPS, Vasudevan J, Pegu B. Group antenatal care: a paradigm shift to explore for positive impacts in resource-poor settings. J Prev Med Public Health. 2021;54:81–4. 33618503 10.3961/jpmph.20.349 PMC7939754

[7] Lori JR, Kukula VA, Liu L, et al. Improving health literacy through group antenatal care: results from a cluster randomized controlled trial in Ghana. BMC Pregnancy Childbirth. 2024;24:37. 38182969 10.1186/s12884-023-06224-x PMC10768124

[8] Zielinski R, Kukula V, Apetorgbor V, et al. “With group antenatal care, pregnant women know they are not alone”: the process evaluation of a group antenatal care intervention in Ghana. PLoS ONE. 2023;18:e0291855. 37934750 10.1371/journal.pone.0291855 PMC10629640

[9] Grenier L, Suhowatsky S, Kabue MM, et al. Impact of group antenatal care (G-ANC) versus individual antenatal care (ANC) on quality of care, ANC attendance and facility-based delivery: a pragmatic cluster-randomized controlled trial in Kenya and Nigeria. PLoS ONE. 2019;14:e0222177. 31577797 10.1371/journal.pone.0222177 PMC6774470

[10] Patil CL, Klima CS, Steffen AD, et al. Implementation challenges and outcomes of a randomized controlled pilot study of a group prenatal care model in Malawi and Tanzania. Int J Gynaecol Obstet. 2017;139:290–6. 28905377 10.1002/ijgo.12324 PMC5673548

[11] Noguchi L, Grenier L, Kabue M, et al. Effect of group versus individual antenatal care on uptake of intermittent prophylactic treatment of malaria in pregnancy and related malaria outcomes in Nigeria and Kenya: analysis of data from a pragmatic cluster randomized trial. Malar J. 2020;19:51. 31996209 10.1186/s12936-020-3099-x PMC6990503

[12] Liese KL, Kapito E, Chirwa E, et al. Impact of group prenatal care on key prenatal services and educational topics in Malawi and Tanzania. Int J Gynecol Obstet. 2021;153:154–9. 10.1002/ijgo.13432 PMC8073885 33098114

[13] Jeremiah RD, Patel DR, Chirwa E, et al. A randomized group antenatal care pilot showed increased partner communication and partner HIV testing during pregnancy in Malawi and Tanzania. BMC Pregnancy Childbirth. 2021;21:790. 34819018 10.1186/s12884-021-04267-6 PMC8611988

[14] Sharma J, O’Connor M, Rima Jolivet R. Group antenatal care models in low- and middle-income countries: a systematic evidence synthesis. Reprod Health. 2018;15:38. 29506531 10.1186/s12978-018-0476-9 PMC5836451

[15] Lundeen T, Musange S, Azman H, et al. Nurses’ and midwives’ experiences of providing group antenatal and postnatal care at 18 health centers in Rwanda: a mixed methods study. PLoS ONE. 2019;14:e0219471. 31295335 10.1371/journal.pone.0219471 PMC6622527

[16] Harsha Bangura A, Nirola I, Thapa P, et al. Measuring fidelity, feasibility, costs: an implementation evaluation of a cluster-controlled trial of group antenatal care in rural Nepal. Reprod Health. 2020;17:5. 31952543 10.1186/s12978-019-0840-4 PMC6967133

[17] Suhowatsky S, Onguti B, Ae Apetorgbor V, et al. Implementing group antenatal care: the global experience. Pract Midwife. 2024;27:46–9.

[18] Sadiku F, Bucinca H, Talrich F, et al. Maternal satisfaction with group care: a systematic review. AJOG Glob Rep. 2024;4:100301. 38318267 10.1016/j.xagr.2023.100301 PMC10839533

[19] Jolivet RR, Uttekar BV, O’Connor M, et al. Exploring perceptions of group antenatal Care in Urban India: results of a feasibility study. Reprod Health. 2018;15:57. 29615069 10.1186/s12978-018-0498-3 PMC5883286

[20] Hellar A, Kinyina A, Sospeter P, et al. An assessment to inform programming for antenatal care services in six health facilities in Geita Region, Tanzania: a cross-sectional baseline survey. Epub ahead of print 9 September 2024. 10.21203/rs.3.rs-4829306/v1.

[21] The Strengthening the Reporting of Observational Studies in Epidemiology (STROBE) statement: guidelines for reporting observational studies, https://core.ac.uk/reader/33050540?utm_source=linkout (accessed 18 April 2025). 10.1136/bmj.39335.541782.AD PMC2034723 17947786

[22] Pinnock H, Barwick M, Carpenter CR, et al. Standards for Reporting Implementation Studies (StaRI) statement. *BMJ* 2017; i6795. 10.1136/bmj.i6795 PMC5421438 28264797

[23] Hellar AM. Group antenatal care implementation study in Geita, Tanzania (2022–2024) , https://dataverse.harvard.edu/dataset.xhtml?persistentId=doi:10.7910/DVN/RLAJMC (2025, accessed 20 December 2025).

[24] KoboToolBox, www.kobotoolbox.org.

[25] Konje ET, Magoma MTN, Hatfield J, et al. Missed opportunities in antenatal care for improving the health of pregnant women and newborns in Geita district, Northwest Tanzania. BMC Pregnancy Childbirth. 2018;18:394. 30290769 10.1186/s12884-018-2014-8 PMC6173847

[26] Adaji SE, Jimoh A, Bawa U, et al. Women’s experience with group prenatal care in a rural community in northern Nigeria. Int J Gynecol Obstet. 2019;145:164–9. 10.1002/ijgo.12788 PMC6449218 30779108

[27] Mehay A, Motta GD, Hunter L, et al. What are the mechanisms of effect of group antenatal care? A systematic realist review and synthesis of the literature. BMC Pregnancy Childbirth. 2024;24:625. 39354405 10.1186/s12884-024-06792-6 PMC11446066

[28] McKinnon B, Sall M, Vandermorris A, et al. Feasibility and preliminary effectiveness of group antenatal care in Senegalese health posts: a pilot implementation trial. Health Policy Plan. 2020;35:587–99. 32155254 10.1093/heapol/czz178

[29] Noguchi L, Grenier L, Suhowatsky S, et al. Group antenatal care is associated with increased uptake of intermittent preventive treatment of malaria in pregnancy among women in Nigeria, compared to routine antenatal care: secondary analysis of a cluster randomized controlled trial. Am J Obstet Gynecol. 2018;219:640.

[30] Kretchy IA, Atobrah D, Adumbire DA, et al. Enhancing the uptake of intermittent preventive treatment for malaria in pregnancy: a scoping review of interventions and gender-informed approaches. Malar J. 2025;24:49. 39966899 10.1186/s12936-025-05275-z PMC11837586

[31] Gutman JR, Stephens DK, Tiendrebeogo J, et al. A cluster randomized trial of delivery of intermittent preventive treatment of malaria in pregnancy at the community level in Burkina Faso. Malar J. 2020;19:282. 32758233 10.1186/s12936-020-03356-9 PMC7409482

[32] Peterkin T, Eke E, Don-Aki J, et al. Increased uptake of intermittent preventive treatment for prevention of malaria in pregnancy and scale-up of group antenatal care in Nasarawa State. Nigeria Am J Obstet Gynecol. 2024;230:S627.

[33] Hill J, Hoyt J, Van Eijk AM, et al. Factors affecting the delivery, access, and use of interventions to prevent malaria in pregnancy in sub-Saharan Africa: a systematic review and meta-analysis. PLoS Med. 2013;10:e1001488. 23935459 10.1371/journal.pmed.1001488 PMC3720261

[34] Yoder PS, Nsabagasani X, Eckert E, et al. Perspectives of health care providers on the provision of intermittent preventive treatment in pregnancy in health facilities in Malawi. BMC Health Serv Res. 2015;15:354. 26318623 10.1186/s12913-015-0986-x PMC4552981

[35] Lori JR, Ofosu-Darkwah H, Boyd CJ, et al. Improving health literacy through group antenatal care: a prospective cohort study. BMC Pregnancy Childbirth. 2017;17:228. 28705179 10.1186/s12884-017-1414-5 PMC5513199

[36] Peña-Rosas JP, De-Regil LM, Garcia-Casal MN, et al. Daily oral iron supplementation during pregnancy. *Cochrane Database Syst Rev*; 2015. Epub ahead of print 22 July 2015. 10.1002/14651858.CD004736.pub5. 10.1002/14651858.CD004736.pub5 PMC8918165 26198451

[37] Imdad A, Bhutta ZA. Routine iron/folate supplementation during pregnancy: effect on maternal anaemia and birth outcomes. Paediatr Perinat Epidemiol. 2012;26:168–77. 22742609 10.1111/j.1365-3016.2012.01312.x

[38] Campbell OMR, Calvert C, Testa A, et al. The scale, scope, coverage, and capability of childbirth care. Lancet. 2016;388:2193–208. 27642023 10.1016/S0140-6736(16)31528-8

[39] Mohan D, LeFevre AE, George A, et al. Analysis of dropout across the continuum of maternal health care in Tanzania: findings from a cross-sectional household survey. Health Policy Plan. 2017;32:791–9. 28334973 10.1093/heapol/czx005

[40] Klaic M, Kapp S, Hudson P, et al. Implementability of healthcare interventions: an overview of reviews and development of a conceptual framework. Implement Sci. 2022;17:10. 35086538 10.1186/s13012-021-01171-7 PMC8793098

[41] Spetz J, Burgess JF, Phibbs CS What determines successful implementation of inpatient information technology systems? *Am J Manag Care*; 18, https://pubmed.ncbi.nlm.nih.gov/22435909/ (2012, accessed 20 April 2025). 22435909

