# Supplementary material for: Integrating group antenatal care into routine services: a registry-based cohort study in Geita, Tanzania
Source: BMC Glob Public Health. 2026 Feb 2;4:12. doi: 10.1186/s44263-026-00243-4 (PMC12862910; doi:10.1186/s44263-026-00243-4)
Supplement: Supplementary file 1 — Supplementary Material l: StaRI Checklist - Completed Standards for Reporting Implementation Studies checklist with manuscript cross-references. [file 44263_2026_243_MOESM1_ESM.docx]

**
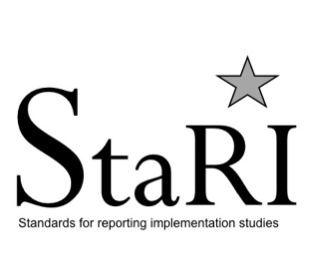
SUPPLEMENTARY MATERIAL 1: Standards for Reporting Implementation Studies: The StaRI checklist**

Manuscript Title: Integrating Group Antenatal Care into Routine Services: A Registry-Based Cohort Study in Geita, Tanzania

| **Checklist item** | | **Reported on page #** | **Implementation Strategy** | **Reported on page #** | **Intervention** |
| --- | --- | --- | --- | --- | --- |
|  | | v | “Implementation strategy” refers to how the intervention was implemented | v | “Intervention” refers to the healthcare or public health intervention that is being implemented. |
| **Title and abstract** | | | | | |
| Title | **1** | 1 | An implementation study; registry-based, observational cohort in Geita Region, Tanzania | | |
| Abstract | **2** | 2 | integrated into routine facilities, readiness assessment, training, supervision) using G-ANC as an intervention with improved outcomes (ANC4+, IPTp3+, facility delivery, birth outcomes) | | |
| **Introduction** | | | | | |
| Background | **3** | 4-5 | Low ANC4+ coverage in Tanzania and delayed ANC initiation; contextual barriers in Geita | | |
| Rationale | **4** | 4 | Implementation strategy was using an MOH-led approach, with a site readiness assessment prior to implementation to understand challenges | 4 | Based on evidence from literature that G-ANC improves ANC uptake and promotes peer support |
| Aims and objectives | **5** | 5 | Aim of the study was to evaluate how G-ANC improves ANC attendance, utilization of services and outcomes in routine facilities | | |
| **Methods: description** | | | | | |
| Design | **6** | 6 | A registry-based, single-group cohort implementation study using a quantitative observational design, conducted across six public health facilities at different health system tiers in both semi-urban and rural areas of Geita Region, Tanzania. The study followed the Standards for Reporting Implementation Studies (StaRI) and STROBE guidelines. | | |
| Context | **7** | 7- 8 | Described the study context, which is the setting in which the study was conducted, Geita region, in routine government facilities under resource constraints but also following national ANC guidelines and WHO 2016 ANC framework | | |
| Targeted ‘sites’ | **8** | 6 | Study setting highlights the 6 facilities where the study was implemented (2 hospitals, 2 health centers, 2 dispensaries based on a pre-implementation site readiness assessment including staffing capacity | 10 | Study population described as all women presenting to G-ANC with GA > 20 weeks. Those with ≤ 20 weeks were offered routine individual ANC until they reached 20 weeks when they were transitioned to G-ANC |
| Description | **9** | 8-9 | Following WHO 2016 ANC recommendations and integrated into government facilities in routine settings, with training, implementation and supportive supervision | 9 | G-ANC cohorts of 8–12 women ≥20 weeks GA, structured curriculum for each session, 5 sessions in total, standard ANC package following national guidelines. |
| Sub-groups | **10** |  | N/A – no sub-groups were recruited for this study | | |
| **Methods: evaluation** | | | | | |
| Outcomes | **11** | 11 | Primary and secondary endpoints defined: ANC4+, IPTp3+, facility delivery, adverse birth outcomes (stillbirth, LBW, preterm, neonatal death). | N/A | There were no pre-specified primary and other outcome(s) of the intervention or any pre-determined targets |
| Process evaluation | **12** | N/A | Not included in this quantitative analysis | | |
| Economic evaluation | **13** | N/A | Not undertaken – not the scope of this paper | N/A | Not undertaken – not the scope of this paper |
| Sample size | **14** | 11 | Full census of eligible women (n=5,936); justification based on projected workload and precision of estimates | | |
| Analysis | **15** | 13-14 | Descriptive statistics; logistic regression; covariates: maternal age, parity, GA at entry, facility level. Limitations of not adjusting for clustering acknowledged | | |
| Sub-group analyses | **16** | 16, 18 | ANC utilization and outcomes stratified by facility level. This is reported in Table 3, and Fig 2 | | |
| **Results** | | | | | |
| Characteristics | **17** | 15 | 5,936 women, 149 cohorts; demographics and GA at entry described. Reported in Table 2 | 15 | Same cohort recruited received the G-ANC intervention |
| Outcomes | **18** | 14-20 | ANC4+ completion, IPTp3+, facility delivery, adverse birth outcomes; regression results reported. Reported in Table 4 | 19 | Same as in implementation strategy |
| Process outcomes | **19** | N/A | This was a quantitative study; process outcomes will be reported in a separate qualitative study | | |
| Economic evaluation | **20** | N/A | Not undertaken in this assessment | N/A | Not undertaken in this assessment |
| Sub-group analyses | **21** | 16,18 | Stratified by facility level; ANC utilization and outcomes compared | | |
| Fidelity/ adaptation | **22** | 7,8 | Fidelity ensured through training/supervision; adaptation done for G-ANC entry at ≥20 weeks GA |  | Fidelity to delivering the core components of intervention (where measured) |
| Contextual changes | **23** | N/A | None reported | | |
| Harms | **24** | N/A | None reported | | |
| **Discussion** | | | | | |
| Structured discussion | **25** | 21-26 | Summarizes findings, strengths and limitations, comparison to literature, careful on causality | | |
| Implications | **26** | 27 | Policy relevance; sustainability; facility-level adaptations needed for scale-up all discussed and added to the conclusion | 27 | Same as in implementation strategy |
| **General** | | | | | |
| Statements | **27** | 29-31 | Ethical approvals (NIMR/HQ/R.8a/Vol.IX/4194), funding acknowledgement (Gates Foundation), data sharing: (de-identified dataset deposited and repository details provided in declarations) and conflict of interest statement all included: | | |
