# Supplementary material for: Integrating group antenatal care into routine services: a registry-based cohort study in Geita, Tanzania
Source: BMC Glob Public Health. 2026 Feb 2;4:12. doi: 10.1186/s44263-026-00243-4 (PMC12862910; doi:10.1186/s44263-026-00243-4)
Supplement: Supplementary file 2 — Supplementary Material 2: STROBE Checklist - Completed STROBE cohort checklist with manuscript cross-references. [file 44263_2026_243_MOESM2_ESM.docx]

**SUPPLEMENTARY MATERIAL 2: STROBE CHECKLIST FOR OBSERVATIONAL COHORT STUDIES**

**Manuscript:** Integrating Group Antenatal Care into Routine Services: A Registry-Based Cohort Study in Geita, Tanzania

**Date:** 20 Dec 2025

| **Item No.** | **STROBE Recommendation** | **Where addressed in manuscript** |
| --- | --- | --- |
| 1 | Title/Abstract: Indicate study design in title/abstract; provide informative, balanced summary. | The title and abstract are structured with background, methods, results and conclusions. The study design has been stated as a quantitative, registry-based observational cohort in Geita, Tanzania. |
| 2 | Background/Rationale: Explain scientific background and rationale. | Background section: describes gaps in routine G‑ANC evidence, the WHO 8 ANC contacts context, and local baseline situation in Geita region. |
| 3 | Objectives: State specific objectives, including any prespecified hypotheses. | In the background section, the final paragraph aims to describe implementation and service coverage; it explores potential associations with adverse outcomes |
| 4 | Study design: Present key elements early in the paper. | The methods section included the study design: quantitative, registry‑based single‑group cohort across six public facilities. |
| 5 | Setting: Describe setting, locations, and relevant dates (recruitment, follow‑up, data collection). | This has been described in methods – study setting and implementation i.e. six facilities (dispensary/health center/hospital) in Geita Region, Tanzania; implementation window of 20 months following routine registry abstraction. |
| 6 | Participants: Give eligibility criteria and sources/methods of selection; follow‑up methods. | The methods describe the participants and eligibility: all eligible ANC clients invited to G‑ANC; ≥20 weeks formal entry; cohorts were formed by similar GA and followed‑up through delivery using a cohort tracker |
| 7 | Variables: Clearly define all outcomes, exposures, predictors, potential confounders, and effect modifiers. | Methods – All key variables & outcomes have been described: definitions for ANC4+, IPTp3+, service indicators; adverse birth outcomes (stillbirth ≥28 weeks; prematurity <37 weeks, etc.). |
| 8 | Data sources/measurement: For each variable, give data sources and assessment methods. | In the Methods section, data collection procedures are explained: routine facility registers for ANC and Labor, a cohort tracker |
| 9 | Bias: Describe efforts to address potential sources of bias. | Described in Methods - data quality & analysis: facility‑level clustering handled via GLMM; limitations discuss selection/confounding (e.g., referral to hospitals) and misclassification. |
| 10 | Study size: Explain how study size was arrived at. | This is explained in Methods—projected implementation target: operational target ( approx. 6,000) based on capacity; no formal power calculation; precision discussed in limitations |
| 11 | Quantitative variables: Explain handling in the analyses. | Methods - Data analysis: binary outcomes; facility level as categorical (dispensary used as reference); gestational age used per definitions; no arbitrary categorization beyond standard cut‑points. |
| 12 | Statistical methods: All methods, including confounding control; subgroup/interaction; missing data; loss to follow‑up; sensitivity analyses. | Data analysis explained in the Methods: binomial–logit GLMM with random intercept for facility; adjusted odds ratios with 95% CIs; clustering by facility addressed; denominators specified by indicator and facility level; missingness handled via complete‑case from routine registers; assumptions noted. |
| 13 | Participants (Results): Report numbers at each stage; use a flow diagram if applicable. | In Results—participant profile described: counts of enrolled women (n≈5,936) and cohorts (n=149); Table 1 shows facility distribution. |
| 14 | Descriptive data: Characteristics of participants, exposures, and potential confounders; indicate missing data. | Results—Baseline characteristics (Table 1) as applicable) |
| 15 | Outcome data: Report numbers of outcome events or summary measures over time. | Results—Coverage of ANC services (Figure 2) facility-level coverage (Figure 3); adverse outcomes summarized; Table 4 multivariable model. |
| 16 | Main results: Unadjusted and adjusted estimates with precision (e.g., 95% CI); clearly state confounders included. | Results—Table 4: adjusted ORs from GLMM; footnote lists covariates and reference categories; p‑values provided. |
| 17 | Other analyses: Subgroups, interactions, sensitivity analyses. | Results/Discussion: stratification by facility level in Fig 3; sensitivity addressed in narrative; acknowledge data/precision limits. |
| 18 | Key results: Summarize key results with reference to objectives. | Conclusions: high ANC4+ and strong service uptake; described as associations only; |
| 19 | Limitations: Discuss limitations of the study, including sources of potential bias or imprecision. | Discussion section includes limitations: observational design; no causal inference; referral/selection confounding; precision for some estimates limited. |
| 20 | Interpretation: Provide cautious overall interpretation considering objectives, limitations, multiplicity of analyses, and other evidence. | Discussion/Conclusions: interpret as associations within implementation context; aligned with literature; Future research needs were specified. |
| 21 | Generalisability: Discuss external validity of the results. | Discussion: generalizability to similar public‑sector settings when readiness criteria met; notes on system capacity and earlier entry. |
| 22 | Funding: Give source of funding and role of funders. | Declarations—Funding: Gates Foundation support acknowledged; role of funder; implementing partners; competing interests declared; data availability with Harvard Dataverse DOI. |
