# Supplementary material for: Integrating group antenatal care into routine services: a registry-based cohort study in Geita, Tanzania
Source: BMC Glob Public Health. 2026 Feb 2;4:12. doi: 10.1186/s44263-026-00243-4 (PMC12862910; doi:10.1186/s44263-026-00243-4)
Supplement: Supplementary file 3 — Supplementary Material 3: Readiness Assessment Tool - facility readiness assessment instrument to evaluate staffing, infrastructure/equipment, leadership/commitment with scoring guidance. [file 44263_2026_243_MOESM3_ESM.docx]

**SUPPLEMENTARY MATERIAL 3: HEALTH FACILITY READINESS ASSESSMENT TOOL**

Manuscript Title: Integrating Group Antenatal Care into Routine Services: A Registry-Based Cohort Study in Geita, Tanzania

**Background information**

The “Mlinde Mama” project is planning to implement its activities in five selected health facilities in Geita region to pilot a digital health intervention that will enhance care through facilitating clinical decision making and hence and reduce adverse birth outcomes during antenatal care by using a group ANC Model as a platform for implementation. Mlinde Mama project will leverage on availability of prior trained GANC trainers cum mentors, GANC trained HC providers and equipment such as BP machines and weighing scales that were provided to support GANC study implementation.

To achieve this, “Mlinde Mama” project staff will conduct rapid facility assessment to gauge the status of health facilities’ readiness to implement Mlinde Mama project through the GANC platform and supported with Machine Learning (ML.)

**Purpose**

The information collected will help Mlinde Mama project to select the sites for the pilot study and make preparations for the implementation including training plans, procurement plans as well as advocacy for availability of commodities and supplies for smooth operation of this project.

**Method for health facility assessment**

The assessment will be done through site visit and collection of information using a structured questionnaire which will include asking a few questions to the health facility in charge/ designee and visual observation.

**The key areas to be assessed are;**

1. Provision of routine ANC services.
2. Availability of space for provision of GANC services.
3. Availability of trainers/mentors and health care providers for GANC.
4. Availability of standard ANC services.
5. Availability of equipment, commodities and supplies for standard ANC.
6. Availability of HMIS tools and number of pregnant women attending first ANC before 24 weeks of gestation age.
7. Infection, Prevention and Control (IPC) and PPE.

**Health Facility Readiness Assessment Tool**

**Health Facility Identification**

| **Health Facility Name** | **Responses Code** |
| --- | --- |
| **District** |  |
| **Facility Level** | District Hospital…………..2  Health Center…………….3  Dispensary………………...4 |
| **Name of Assessor** |  |
| **Date (dd/ mm/yyyy)** |  |

**Section A: Provision of routine ANC services**

|  | **Question** | **Responses Code** |
| --- | --- | --- |
| ***A1*** | Does this facility offer routine antenatal care services? | **Yes ……………1**  **No……………..2** |
| ***A2*** | How many days in a week does this facility provide ANC services? | **#..........................**  **Days (Mention) ………………………………………** |
| ***A3*** | How many skilled ANC providers are working at ANC clinic on the day of service? | **#..........................** |

**Section B: Availability of space for provision of GANC services**

|  | **Question** | **Responses Code** | | |
| --- | --- | --- | --- | --- |
|  | **Space for GANC meeting** |  | | |
| ***B1*** | Currently is there a place in this facility where a group meeting for up to 15 pregnant women can meet? | **Yes available …………………….1**  **Not available……………………..2**  **Could be made available……….3** | | |
| ***B2*** | Are there benches. chairs enough for sitting up to 15 people. | **Yes available …………………….1**  **Not available……………………...2**  **Could be made available………..3** | | |
|  |  | **Yes** | **No** | |
| ***B3*** | Availability of toilet near the GANC space. | 1 | 2 | |
| ***B4*** | Availability of hand washing facilities | 1 | 2 | |
| ***B5*** | Availability of safe drinking water | 1 | 2 | |
| ***B6*** | Is there a place for data storage for GANC study records? | 1 | 2 | |
| ***B7*** | Is the place secure?/ have locked cabinets? | 1 | 2 | |
|  | **Examination Room** |  | | |
| ***B8*** | Is there a room adjacent to the meeting room for individual consultation and examination? | Yes available ……………………...….1  Not available……………………..…....2  Could be made available…………...….3 | | |
| ***B9*** | If examination room is available, observe for visual and auditory privacy. | Visual and auditory privacy available …1  Visual privacy only……………………..2  No privacy …………………………….3 | | |
| ***B10*** | Does the examination room have the following? | Yes | | No |
| ***B10a*** | Examination gloves? | 1 | | 2 |
| ***B10b*** | Sharps container? | 1 | | 2 |
| ***B10c*** | Alcohol hand rub? | 1 | | 2 |
| ***B10d*** | Waste receptacle with lid and liner? | 1 | | 2 |
| ***B10e*** | Liquid soap for hand washing ? | 1 | | 2 |

**Section C: Availability of Trainers/Mentors/Health Care Providers for GANC**

|  | **Question** | **Response Code** |
| --- | --- | --- |
| ***C1a*** | Is there a health care provider at this District who has been trained on GANC as a Trainer/Mentor? (***ask the district RCHCo or NO***) | Yes………………1  No……………….2  Don’t know…….88 |
| ***C1b*** | If Yes, How many? | **#.......................** |
| ***C2a*** | Is there a health care provider at this HF who has been trained on GANC? (A***sk the HF In-charge/ patron/matron***) | Yes………………1  No……………….2  Don’t know…….88 |
| ***C2b*** | If Yes , How many? | **#.........** |
| ***C2c*** | Is she/he/they still working at the same district? | Yes………………1  No……………….2  Don’t know…….88 |
| ***C2d*** | Is she/he/they still working at the HF? | Yes………………1  No……………….2  Don’t know…….88 |
| **C3** | **Cadre of trained HCP trained on GANC** | **#** |
| ***C3a*** | Obstetrician/Gynaecologist |  |
| ***C3b*** | Medical Officer |  |
| ***C3c*** | Assistant Medical Officer |  |
| ***C3d*** | Clinical officer |  |
| ***C3e*** | Assistant Clinical Officer |  |
| ***C3f*** | Nursing Officer |  |
| ***C3g*** | Assistant Nursing Officer |  |
| ***C3h*** | Medical Attendant |  |
| ***C3i*** | Other (specify) |  |

**Section D: Availability of standard ANC services**

| D | Is [*read service*] routinely conducted for all antenatal care clients as per national standards? | Responses Code | |  |
| --- | --- | --- | --- | --- |
|  |  | Yes | No | Not always/ explain |
| ***D1*** | Weighing clients | 1 | 2 |  |
| ***D2*** | Taking blood pressure | 1 | 2 |  |
|  | ***Lab Tests*** |  |  |  |
| ***D3*** | Urine test for protein | 1 | 2 |  |
| ***D4*** | Blood test for Hb estimation | 1 | 2 |  |
| ***D5*** | Blood test for HIV | 1 | 2 |  |
| ***D6*** | Blood test for syphilis | 1 | 2 |  |
| ***D7*** | Malaria testing | 1 | 2 |  |
|  | ***Other investigations*** |  |  |  |
| ***D8*** | Ultra sound | 1 | 2 |  |
| ***D9*** | Blood grouping | 1 | 2 |  |
| ***D10*** | Test for Rh factor | 1 | 2 |  |
|  | ***Other services*** |  |  |  |
| ***D11*** | Tetanus toxoid vaccination |  |  |  |
|  | ***Health education and Counselling*** |  |  |  |
| ***D12*** | Conducting group health education sessions | 1 | 2 |  |
| ***D13*** | Breastfeeding and nutritional counsel | 1 | 2 |  |
| ***D14*** | Counselling for FP | 1 | 2 |  |

**Section E: Availability of Equipment/Commodities and Supplies for Standard ANC**

| **E** | **Equipment and Testing Supplies** | **Responses Code** | | | |
| --- | --- | --- | --- | --- | --- |
|  |  | **Yes available** | **Not available** | **Functioning** | **Not functioning** |
| ***E1a*** | Blood pressure apparatus | 1 | 2 | 1 | 2 |
| ***E1b*** | Stethoscope | 1 | 2 | 1 | 2 |
| ***E1c*** | Fetal stethoscope (Fetoscope) | 1 | 2 | 1 | 2 |
| ***E1d*** | Doppler | 1 | 2 | 1 | 2 |
| ***E1e*** | Adult weighing scale | 1 | 2 | 1 | 2 |
| ***E1f*** | Height scale | 1 | 2 | 1 | 2 |
| ***E1g*** | Measuring tape | 1 | 2 |  |  |
| ***E1h*** | Blanket or sheet to cover women |  |  |  |  |
| ***E1i*** | Needles and syringes (2cc,5cc,10cc,20cc) |  |  |  |  |
| ***E1j*** | Tourniquet | 1 | 2 |  |  |
| ***E1k*** | Tendon hammer | 1 | 2 | 1 | 2 |
| ***E1l*** | Wall Clock | 1 | 2 | 1 | 2 |
| ***E1m*** | Vaginal speculum | 1 | 2 | 1 | 2 |
| ***E1n*** | Infection prevention (IPs) supplies – sharps box; waste receptacle; | 1 | 2 |  |  |
| ***E1o*** | IV giving sets | 1 | 2 |  |  |
| ***E1p*** | Cuvet | 1 | 2 |  |  |
| ***E1q*** | Hemocue machine | 1 | 2 | 1 | 2 |
| ***E1r*** | vacutainer bottles for blood collection | 1 | 2 |  |  |
| ***E1s*** | LLINs | 1 | 2 |  |  |
| ***E1t*** | Examination bed | 1 | 2 | 1 | 2 |
| ***E1u*** | Screens for privacy | 1 | 2 | 1 | 2 |
| ***E1v*** | Multistix/Uristix | 1 | 2 |  |  |
| ***E1w*** | Glucometer | 1 | 2 | 1 | 2 |
|  |  |  |  |  |  |
| ***E2*** | ***Medications/ Vaccine*** |  |  |  |  |
| E2a | Iron and/or folic acid | 1 | 2 |  |  |
| E2b | Tetanus toxoid vaccine | 1 | 2 |  |  |
| E2c | Mebendazole/Albendazole | 1 | 2 |  |  |
| E2d | Calcium | 1 | 2 |  |  |
| E2e | Mg2SO4 | 1 | 2 |  |  |
|  |  |  |  |  |  |
| ***E3*** | ***Anti – hypertensives*** |  |  |  |  |
| E3a | Methyldopa | 1 | 2 |  |  |
| E3b | Nifedipine | 1 | 2 |  |  |
| E3c | Labatolol | 1 | 2 |  |  |
| E3d | Hydralazine | 1 | 2 |  |  |
|  | IV fluids | 1 | 2 |  |  |
| ***E4*** | ***Antimalarials*** |  |  |  |  |
| E4a | SP tablets | 1 | 2 |  |  |
| E4b | ALU tablets | 1 | 2 |  |  |
| E4c | Artesunate injections | 1 | 2 |  |  |
|  |  |  |  |  |  |

**Section F: Availability HMIS tools and Number of pregnant women attending first ANC before 24 weeks of Gestation Age**

|  | Question | Responses Code | |
| --- | --- | --- | --- |
|  |  | Yes | No |
| F | Does the facility have up to date HMIS tools ( version 2021) |  |  |
| ***F1*** | Antenatal care register | 1 | 2 |
| ***F2*** | Family planning register | 1 | 2 |
| ***F3*** | Postpartum family planning register | 1 | 2 |
| ***F4*** | Discharge register | 1 | 2 |
| ***F5*** | Referral register (general) | 1 | 2 |
| ***F6*** | Obstetric referral register | 1 | 2 |
| ***F7*** | Mortality/death register | 1 | 2 |
| ***F8*** | Labour & Delivery register | 1 | 2 |
| ***F9*** | Postnatal Care register | 1 | 2 |
| ***F10*** | Is the RCH card #4 or maternal booklet available | 1 | 2 |

| ***Request for the ANC register. Using these registers, provide the information Requested Below for the Prior 3 Complete Months*** | | | |
| --- | --- | --- | --- |
| **STATISTICS 2022** | **July** | **August** | **September** |
| Number of women attending ANC before 24 weeks gestational age |  |  |  |

**Section G: IPC and PPE**

|  | **Question** | **Yes** | **No** |
| --- | --- | --- | --- |
| ***G1*** | Does facility have running water stations with soap for hand wash before entered the facility? |  |  |
| ***G2*** | Does Providers have and use hand sanitizer at facility during service provision? |  |  |
| ***G3*** | Does facility have enough stock of mask? (Ask if extra boxes available for each Provider) |  |  |
| ***G4*** | Do providers wear masks? |  |  |
